# Management of a female with recurrence of fibromatosis of the chest wall adjacent to the breast: a case report

**DOI:** 10.1186/1749-8090-8-41

**Published:** 2013-03-08

**Authors:** Cheng Shen, Yubin Zhou, Guowei Che

**Affiliations:** 1 Department of Thoracic Surgery, West-China Hospital, Sichuan University, Chengdu, 610041, China

**Keywords:** Aggressive fibromatosis, Recurrent desmoid tumour, Chest wall, Breast

## Abstract

Extra-abdominal desmoid tumor is a rare soft tissue tumor that is histologically benign, but may behave aggressively. This case report specifically describes the clinical, radiographic, and pathologic features of 27 year-old female who experienced a post-surgical recurrence of fibromatosis of the chest wall over a two-year period of time secondary to previous inadequate excision. The fibromatosis was found to be involving the lower-inner quadrant of her right breast and causing worsening pain. A surgical management strategy was successfully undertaken.

## Background

Desmoid tumor is an aggressive fibromatosis that may occur in abdominal and extra-abdominal areas [1]. They constitute less than 0.03% of all neoplasms [2]. For extra-abdominal desmoids, local recurrence rates range from 24% to 77% in reported series [3-5]. Although the fibromatosis of chest wall represents 8-10% of all cases and surgery is the primary treatment modality, there remains a significant lack of agreement amongst surgeons on how to manage the disease that is suspected to have accompanied involvement of adjacent breast especially for an unmarried woman. We report a case where the tumour had a repeated recurrence over a 2-year period.

### Case presentation

A 27 year-old unmarried female presented to our hospital with worsening pain of the right chest wall region and a recurrent obvious mass adjacent to the lower-inner quadrant of her right breast for 5 months. Two years prior to her current presentation, she presented to a local hospital with a palpable right chest wall mass close to the same quadrant of right breast and underwent a surgical resection. The histological examination showed a typical picture of an aggressive fibromatosis.

**Figure 1:**
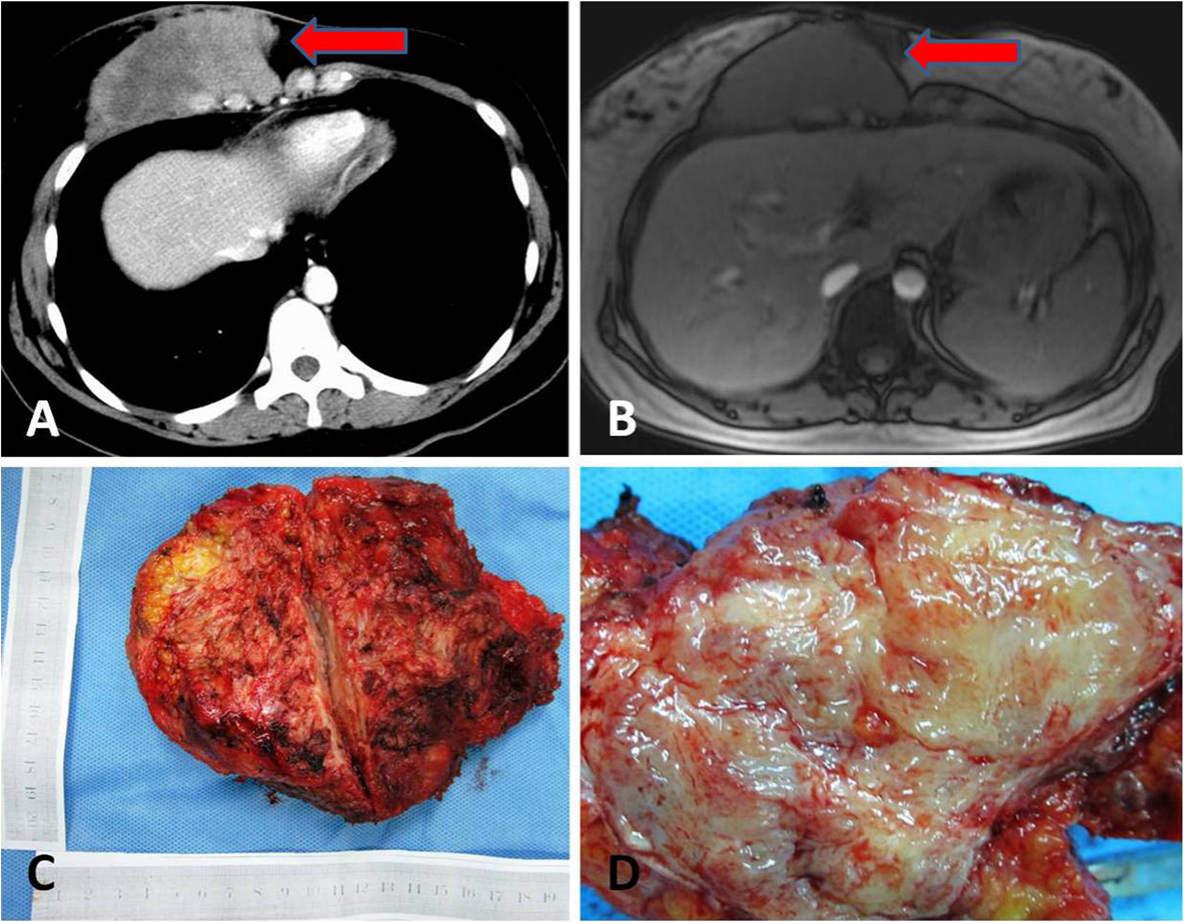
**CT and MRI showed a mass on the right inferior chest wall and the general view of the specimen. A**: Computed tomography showed a 10×5 cm mass on the right inferior chest wall that appeared to be in continuity with the right pectoralis major muscle and right rectus abdominis muscle. **B**: Magnetic Resonance Imaging of the breast and the chest confirmed a 9×4 cm mass in close proximity to the lower-inner quadrant of the right breast, with contact to the ribs. **C**: The surgically removed specimen was lobulated and 14x16x10 cm in size, containing a 10x12x8 cm tumor. **D**: Bisected tumor showed a grossly circumscribed tumor with white, whorled patterns and without necrosis.

Physical examination showed a 15 cm and well-healed surgical scar along the inferior-inner aspect of her right inframammary fold. Plain and contrast-enhanced chest and epigastrium computed tomography (CT) showed a 10×5 cm mass on the right inferior chest wall that appeared to be in continuity with the right pectoralis major muscle and right rectus abdominis muscle (Figure 1A). Magnetic Resonance Imaging (MRI) of the breast and the chest confirmed a 9×4 cm mass in close proximity to the lower-inner quadrant of the right breast, with contact to the ribs (Figure 1B).

As diagnosis was established, surgery was scheduled. Under general anesthesia with selective intubation, the patient lay on the operating table with supine position. She underwent an en bloc resection of the tumor and the underlying musculature (inferior lateral portion of the right pectoralis major muscle and superior portion of the right rectus abdominis musculature, and anterior portion of the right latissimus dorsi muscle) and en bloc resection of the underlying chest wall structures (seventh rib and intercostal muscle). The defect on the right chest wall was then closed with a 10×10 cm Dacron patch. Although the mass was in close proximity to the lower-inner quadrant of the right breast, all surgical margins were negative with the intraoperative frozen section and the right mastectomy was avoided. Then the right breast was closed in the skin flap plasty. Patient was transferred to the intensive care unit and moved to general care after 24 hours and she was discharged to home after a week. She is currently under follow-up.

**Figure 2:**
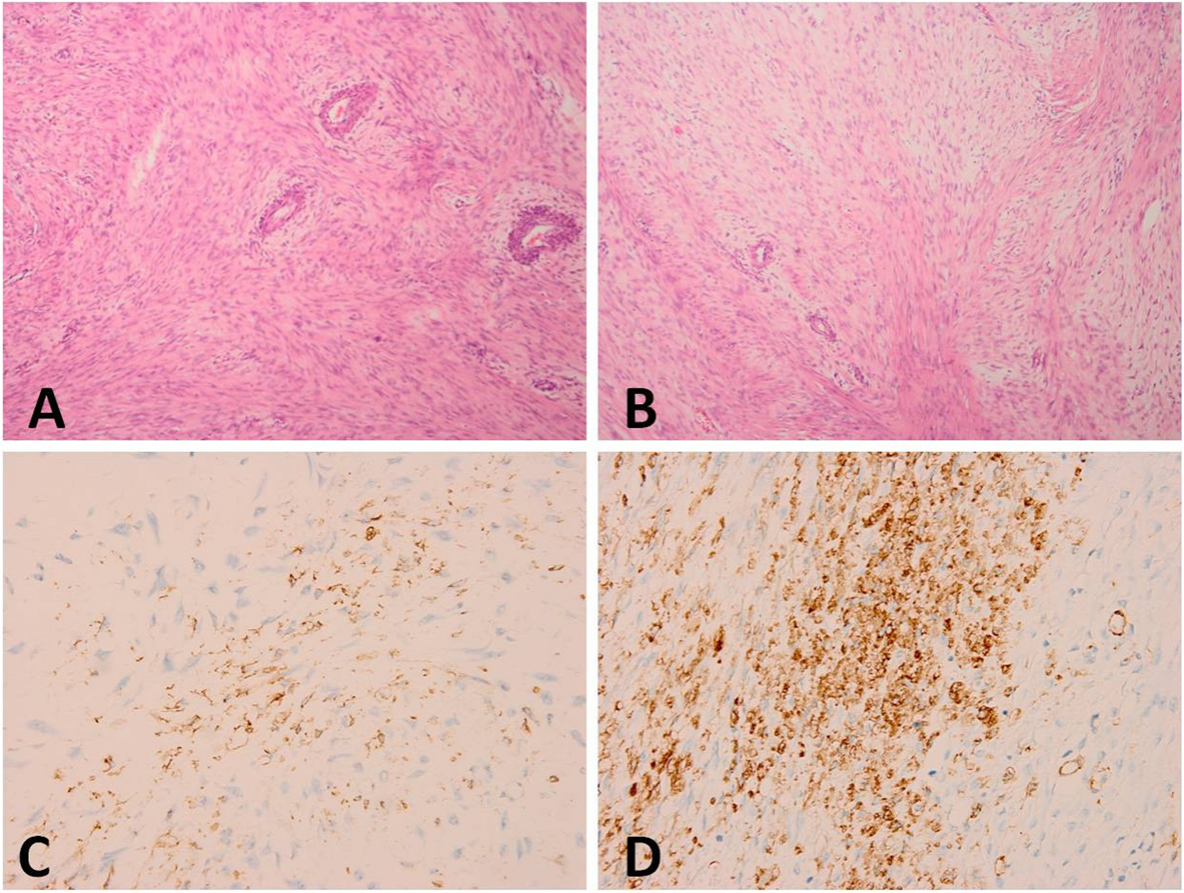
**Histological features.** Section stained with hematoxylin and eosin reveals evenly spaced plump spindle cells arranged in intersecting fascicles and associated with mild to moderate amounts of collagen resembling keloid (**A**, **B** original magnification ×100). On immunohistochemical staining, the spindle cells were positive for muscle-specific actin (MSA) (**C**, original magnification ×400) and smooth muscle actin (SMA) (**D**, original magnification ×400).

The surgically removed specimen was lobulated and 14×16×10 cm in size, containing a 10×12×8 cm tumor. Macroscopically, the bisected tumor showed a grossly circumscribed firm tumor with white, whorled patterns and without necrosis (Figure 1C, D). Microscopically, the lesion was composed of evenly spaced plump spindle cells arranged in intersecting fascicles and associated with mild to moderate amounts of collagen resembling keloid (Figure 2A, B). On immunohistochemical staining, the spindle cells were negative for S-100 protein and Epithelial Membrane Antigen (EMA). The spindle cells were positive for muscle-specific actin (MSA) (Figure 2C) and smooth muscle actin (SMA) (Figure 2D). The histology and immunohistochemical staining supported a diagnosis of fibromatosis (desmoid tumor).

## Conclusions

Extra-abdominal desmoids are frequently referred to as fibromatoses that are mesenchymal neoplasms and develop from the connective tissues, fasciae and aponeuroses.

For the treatment of this recurrent tumour, an aggressive surgical resection strategy with a safe margin (2–3 cm) remains the standard therapeutic anoeuvre, which has been strongly advocated by surgeons dealing with fibromatosis of the chest wall that involves the breast [6,7]. The reasons that support this aggressive surgical approach include the potentials of fibromatosis to grow aggressively locally and to invade into the surrounding structures, thus having a high rate of local recurrence when incompletely excised with positive surgical margins. Some surgeons, however, disagree with such a wide excision and prefer more conservative resection [8-10]. Their concerns are about the less optimal cosmetic outcome and the risk of loss of function. Further, there have not been any reports showing fibromatosis can metastasize. Presence of residual tumor cannot be clearly shown to impact disadvantageously on five-year disease free or overall survival [3]. In our case, we chose to perform a wide en bloc resection to avoid future recurrence, based on negative frozen section on the surgical margin. Thus, we were able to avoid mastectomy. A flap plasty and cosmetology of breast were performed. This is particularly beneficial to the young woman who was going to get married in the next year and her quality of life was not adversely affected.

For a large recurrent fibromatosis involving the chest wall and breast, wide en bloc resection is necessary to prevent future recurrence but shall avoid mastectomy if possible. This surgical management is particularly useful for unmarried young women.

## Consent

Written informed consent was obtained from the patient for publication of this case report and any accompanying images. A copy of the written consent is available for review by the Editor-in-Chief of this journal.

## Competing interests

The authors declare that they have no competing interests.

## Authors’ contributions

CS was involved in drafting the manuscript. YZ was involved in acquisition of data and preparing the figures. GC designed and revised the manuscript. All authors have read and approved the final manuscript.
